# Staying in or out? COVID-19-induced healthcare utilization avoidance and associated socio-demographic factors in rural India

**DOI:** 10.1186/s12889-023-16282-7

**Published:** 2023-07-27

**Authors:** Michael Safo Oduro, Prince Peprah, Anthony Kwame Morgan, Williams Agyemang-Duah

**Affiliations:** 1 Pfizer, Inc., Pharm Sci and PGS Statistics, 445 Eastern Point Rd, Groton, Connecticut USA; 2 Social Policy Research Center, UNSW, Sydney, Australia; 3 Center for Primary Health Care and Equity, UNSW, Sydney, Australia; 4 Department of Geography and Rural Development, Kwame Nkrumah University of Science and Technology, Kumasi, Ghana; 5 Department of Geography and Planning, Queen’s University, K7L 3N6 Kingston, Ontario Canada

**Keywords:** COVID-19, Socio-economic factors, Prevalence, Patient acceptance of healthcare, Logistic models, India

## Abstract

**Background:**

Although evidence on healthcare utilization avoidance during COVID-19 pandemic is emerging, such knowledge is limited in rural settings. An effective policy to the COVID-19 shocks and stresses in rural settings require empirical evidence to inform the design of health policies and programmes. To help overcome this evidence gap and also contribute to policy decisions, this study aimed at examining COVID-19-induced healthcare utilization avoidance and associated factors in rural India.

**Methods:**

This study used the third-round data from the COVID-19-Related Shocks in Rural India survey conducted between 20-24 September, 2020 across six states. The outcome variable considered in this study was COVID-19-induced healthcare utilization avoidance. Multivariable Binary Logistic Regression Model via Multiple Imputation was used to assess the factors influencing COVID-19-induced healthcare utilization avoidance.

**Results:**

Data on 4,682 respondents were used in the study. Of this, the prevalence of COVID-19-induced healthcare utilization avoidance was 15.5% in rural India across the six states. After adjusting for relevant covariates, participants from the Bihar State have significantly higher likelihood of COVID-19-induced healthcare utilization avoidance compared to those from the Andhra Pradesh. Also, participants whose educational level exceeds high school, those who use government hospital/clinic, engage in daily wage labour in agriculture have significantly higher odds of COVID-19-induced healthcare utilization avoidance compared to their counterparts.

**Conclusion:**

Our study revealed that state of residence, type of health facility used, primary work activity and educational level were associated with COVID-19-induced healthcare utilization avoidance in rural India. The findings suggest that policy makers and public health authorities need to formulate policies and design interventions that acknowledge socioeconomic and demographic factors that influence healthcare use avoidance.

## Introduction

Pandemics have always been part of human existence [1]. The prevailing devastating effects of the COVID-19 pandemic are not significantly different from pandemics that visited the world in previous times, albeit the effects of COVID-19 are more widespread than most of the earlier ones that plagued the world [2, 3]. Within the healthcare delivery fraternity, for instance, effects are pronounced; stemming from a shift from non-COVID-19-related care and non-life-threatening sicknesses to COVID-19-related care. Beyond that, citizens globally were encouraged to defer or delay, if possible, non-COVID-19-related care for adequate attention to be accorded to the fight against the COVID-19 pandemic; through the reduction in human contacts and the diversion of resources (both material and human) to the testing, and treatment of COVID-19 patients [1, 4, 5]. In part, these measures, coupled with the fear of contracting the virus if one moves out of their homes (especially in the height of the pandemic, where vaccines were nowhere near) have somehow altered health behaviour in relation to formal healthcare use [1, 6–10]. Hebbar et al. [1] in their probe into the prospects and challenges to healthcare delivery during the COVID-19 pandemic in India observed instances of deferred and delayed healthcare use. These were consequences of both reconfiguration of healthcare delivery (focusing on life threatening or severe cases) and intentional decisions by patients for fear of contracting the virus either at the healthcare centres or on their way to such centres. The extent of these delayed and deferred healthcare seeking behaviour must interest health researchers and health policy makers alike.

Evidence from past epidemics like the Ebola Epidemic in West Africa, according to Elston, Cartwright and Ndumbi (2017) [11], shows that indirect effects of catastrophic events tend to have more deleterious effects than the direct effects since the former is often overlooked. For instance, the Ebola Epidemic in West Africa led to restricted healthcare access, breakdown in trust for health services and a general decline in the utilization of healthcare significantly, particularly among women and children.

To this problem, the healthcare system responded by increasing telehealth and telemedicine services [12] to offset some of the anticipated long-term consequences of delayed in-person care at the onset of the COVID-19 pandemic. However, detection of some health conditions may require a physical examination, rather than remotely monitoring patients. However, the adoption of telemedicine is variably influenced by age, educational attainment and other factors including locational attributes [4, 13]. For rural residents also, the prospects of telemedicine are limited, implying that the deferral of in-person visits could have much more unprecedented consequences.

By enabling remote patient-provider communication and remote access to specialists for consultation, telemedicine programs can help overcome transportation challenges in geographically dispersed rural areas [14, 15]. However, significant obstacles to telemedicine adoption among rural residents exist and include a lack of access to the necessary broadband internet [16, 17], limited access to technology at home, low digital literacy, and skepticism about telemedicine as a practical health service [18]. Furthermore, constraints like a lack of community healthcare providers’ ability to communicate health information and a lack of patient involvement capabilities have been noted [13].

The deleterious effects of delayed care have been observed in the wake of natural disasters [19, 20]. Furthermore, deferred care during the COVID-19 pandemic may already be leading to increased morbidity and mortality in many communities [21–24]. This threatens to be particularly acute among disadvantaged communities.

Studies in other jurisdictions have provided evidence of reduced formal healthcare use. For illustration, a sample of 2,314 residents of St. Louis County, Missouri, the USA, aged above 18 years reported a 53.9% cancellation of healthcare appointments-either by the patient or by care provider [8]. Dental services (31.1%) and primary care (22.1%) were the most common care that was deferred or cancelled. Regarding the predictors, being white, an older adult (≥ 65 years old), being a female, having a fair or poor health status, having health insurance, and having more than one medical condition was associated with higher healthcare deferral. Again, Cantor et al. (2022) [6] observed that the COVID-19 prevention measures were associated with reductions in the use of preventive care, elective care, and the number of weekly visits to physician offices, hospitals and other healthcare-related industries. For specialized urology services in Germany, Harke et al. [25] found an 11% reduction in patronage which was attributed to the COVID-19 pandemic. Elsewhere, a 32% decrease in hospital admissions during weeks 11 to 36 in 2020 was reported in the USA [26]. In Rotterdam, Splinter et al. [27] found that up to 20.2% of sampled respondents reported having avoided healthcare, with older age, female sex, low educational level, poor self-appreciated health, unemployment, smoking, concern of contracting COVID-19, and depressive symptoms in addition to experience of anxiety being the associated factors.

While the literature on healthcare avoidance during the COVID-19 pandemic is emerging, such knowledge is quite lacking among rural areas, particularly in developing countries. Such knowledge (COVID-19-induced healthcare utilization avoidance and associated socio-demographic factors) is important as far as the development and the implementation of policies and programmes to promote healthcare use during and after COVID-19 in rural areas is concerned. India has a significantly high population of rural residents, who cannot necessarily benefit from the telemedicine switch that is somehow being used to avert the effects of delayed and deferred non-COVID-19 care. Finally, evidence from other jurisdiction, although can be useful, are limited in terms of peculiarities relating to context, necessitating a study that examines the issue within the Indian context.

Why India? The COVID-19 pandemic in India has created problems on several fronts: the lockdown, while necessary, has affected people differently, with some being much worse off than others; the restructuring of hospital care in response to COVID-19 has forced many patients with non-COVID-19 conditions to delay receiving treatment [1]. A study conducted by Nilima et al. [28] revealed that individuals who expressed concerns about their family’s health were more likely to adhere to the lockdown measures implemented in 28 states and 8 union territories of India. This finding indicates that the perceived risk to the health and well-being of their loved ones played a significant role in influencing people’s compliance with the lockdown measures; with this possibly influencing individuals’ healthcare-seeking behavior, leading to potential avoidance of healthcare services.

In their Situation Update Report, the World Health Organisation (WHO) provided a comprehensive summary of the COVID-19 situation in India with a total of 5,992,532 confirmed COVID-19 cases and 94,503 total deaths. With a high population of rural residents with the earlier attendant challenges discussed [29, 30], high COVID-19 rates and prevention measures of lockdowns [30], India provides the ideal situation for investigation into COVID-19 healthcare utilization avoidance and associated socio-demographic characteristics.

India’s diverse socio-demographic landscape, with variations in education, income, access to healthcare, and cultural factors, offers a unique context to explore the associated factors contributing to healthcare utilization avoidance during the pandemic. By conducting the study in rural India, we shed light on the specific socio-demographic determinants of COVID-19-induced healthcare avoidance in this population, with prospects for developing targeted interventions and policy recommendations to address the issue effectively and for future pandemics. Again, COVID-19 has highlighted the influence of geographic region on the prevalence of infectious diseases. The disease has spread unevenly across different regions, with varying levels of severity and transmission rates [31] among and within regions. Linked to this, variation in population density in India between urban and rural areas, with higher densities observed in urban regions compared to rural areas contributes to the spread of infectious diseases, particularly in urban areas where close proximity and high interaction among individuals facilitate rapid transmission. However, rural areas are significantly diverse than homogeneous, hence, we included spatial data with regard to the state of residence of the participants to establish how variations in locational attributes influence healthcare utilization avoidance.

To achieve these objectives, we used data from round three of the COVID-19-Related Shocks Survey in Rural India to investigate the prevalence of COVID-19-induced healthcare utilisation refusal and the associated factors. The study is founded on the much attention accorded to the direct health effects of COVID-19 such as deaths and reduction in average life expectancy [8, 32].

The prevalence of healthcare use refusal was estimated while the state of residence, gender, educational attainment, receipt of government support, religious affiliation, and primary work were used as predictors. The findings of this study could be used to inform future pandemic preparation planning to reduce healthcare service interruption and excess morbidity and mortality when healthcare resources are limited especially in rural areas.

## Data and methods

### Survey data and sampling procedure

This study used the third-round data from the COVID-19-Related Shocks in Rural India survey conducted between 20-24, September 2020 [33]. Participants included rural residents primarily involved in agricultural activities. The survey was conducted during the period where COVID-19 was fast spreading in India to understand the various impacts of the pandemic in rural India to inform the design and implementation of effective policy response to the COVID-19 related shocks. The survey was conducted by the World Bank, IDinsight, the Development Data Lab and John Hopkins University, however the World Bank remains the primary investigators. The survey covered six major states including Jharkhand, Rajasthan, Uttar Pradesh, Andhra Pradesh, Bihar, and Madhya Pradesh in India [33]. These states were selected for the survey because of their size and diversity in terms of population and economic activity. As a result, their COVID-19 experiences could serve as a useful benchmark for understanding the pandemic impact. Data collection was conducted through the Computer Assisted Telephone Interview (CATI) software. Thus, the survey was produced through the Data Production and Methods Unit of the Development Data Group, deploying via enumerators’ smartphones. The field enumerators were trained personnel from the respective states selected based on their academic qualification and prior research experience. Field enumerators called the selected participants via mobile phones and recorded their responses. If unreached, surveyors attempted to call back respondents up to 7 times, often seeking explicit appointments for suitable times to avoid non-responses. The phone numbers of the participants were obtained from previous projects implemented in the states in which participants contact details were recorded. The survey did not use a single, unified sampling frame to sample phone numbers. The final sample used for the survey was assembled from prior different sample frames, and the selection of the sample frame varied across states and survey round. The sample frames comprise four existing IDinsight projects, and an impact evaluation of the National Rural Livelihoods project implemented by the Ministry of Rural Development, Government of India, to select a participant [33]. Regarding the third round, the survey attempted to reach 12,600 households but 5,200 households were reached representing a response rate of approximately 55%. Validation and consistency checks were incorporated into the SurveyCTO software to avoid human errors. Surveys were also audio audited by monitors to check for consistency and accuracy of question phrasing and answer recording. Finally, supervisors also randomly back-checked a subset of interviews to further ensure data accuracy. Detailed information about the survey, data, sampling procedures and the data collection techniques can be obtained from the World Bank and other studies [33, 34]. The survey questionnaire was adapted from previous projects in the study areas and was modified to include several questions and modules on health conditions, healthcare access, COVID-19-related knowledge, access to financial relief, migration, income and consumption, and agriculture. For the purpose of this study, we mainly used questions relating to access to healthcare and biodata as well as socio-economic characteristics of household heads for our analysis.

### Study variables

The response variable considered in this study was a dichotomous variable called medical visit avoidance. This variable was defined based on a health related module question regarding visits where respondents were interrogated on the question; “In the past month, have you ever decided to not seek a health service due to coronavirus/COVID-19?”. Medical visit avoidance was defined as a dichotomous measure indicating “No (=(0))” for those in disagreement and “Yes = (1)” for those in agreement with not seeking a health service due to the COVID-19 pandemic respectively. It is important to note that this study was premised on the sample of respondents (4,682) who either responded "Yes" or "No" in view of the response variable considered. Furthermore, both categorical and continuous predictor variables were considered in the study. Inclusive of the categorical study variables were gender, state, educational level,motorcycle transportation access, access to government support (via the Pradhan Mantri Gareeb Kalyan Yojna (PMGKY) scheme), health facility type patronized, primary work activity, and the religious affiliation of the respondents. In addition, age, the number of days worked in a week and average revenue earned by respondents were continuous study variables considered.

### Statistical methodology

To address the objectives of this study, descriptive and inferential statistical techniques are used to analyze the data. Via descriptive measures, we maximize insight into the data by obtaining sample frequencies and percentages related to demographic and socioeconomic attributes of respondents and medical visit avoidance during the Coronavirus pandemic in rural India. The inferential methods used in the study are two-fold. First, the Pearson’s Chi-square test of independence [35] is employed to assess the null hypothesis of no relationship between medical visit avoidance and the other categorical independent study variables(gender, state, educationa level, transportation access, access to government support, health facility type patronized, primary work activity, and the religious affiliation of the respondent) separately. If the *p*-values of the resulting tests are less than a predetermined statistical level of significance ($\alpha =0.05$), then there is the indication of a strong evidence of an association between medical visit avoidance and the other categorical predictors separately. Then, we proceed analyzing the data with a Multivariable Binary Logistic Regression Model via Multiple Imputation. Missing occurrences on study variables are inevitable in cross-sectional studies [36–39] and this current study is not an exception. Missingness result in a substantial loss of information, a reduction in precision of statistical estimates and vitiates the validity of analysis [40]. Thus, the resulting incomplete data require appropriate modeling techniques. A plethora of methods exist for handling missing data. Simple methods ranging from Complete Case analysis (CC), Available case analysis $(\textrm{AC})$ and Last Observation Carried Forward (LOCF) operate under the assumption that the mechanism of missingness is Missing Completely at Random(MCAR) [40, 41]. Complete Case Analysis uses only subject variables having a complete set of observations. The Available Case method uses all available information instead of discarding subjects with missing records. The LOCF method assigns the last observed value as a substitute to all missing values. However, these simple methods, when used to address missingness, lead to a dramatic loss of study sample size and reduces statistical power. Thus, it is not advisable, even under strong assumption of MCAR, to use simple methods because parameter estimate results can be extremely biased [42, 43]. In this vein, to address missing data occurrence in this study, a Multiple Imputation analysis approach, a popular technique for handling missing data [44–46] is adopted.

The Multiple Imputation(MI) technique involves three broad stages. Firstly, the missing values in the data are filled in *S* times to generate *S* multiply imputed complete data sets. These values are generated from a plausible model which is based on a set of parameters drawn from a sampling distribution of the parameter estimates. Secondly, the *S* multiply imputed and complete data sets are analyzed separately. Lastly, estimates resulting from the separate analysis are combined for the statistical inference. The MI procedure is robust and results in valid statistical inferences that properly reflect uncertainty due to missingness [40, 41, 47]. In this study, the first stage of the MI approach, which involves the imputation generating stage, uses an approach called the Multiple Imputation by Chained Equations(MICE) [48, 49]. More broadly, the MICE algorithm generates *S* imputations via a specification of univariate regression models for each variable subject to imputation, conditioned on other variables in the dataset. A distinguishing feature of the MICE proceedure is its potential to handle differing variable types (continuous or categorical variables). It is important to note that the MICE algorithm is premised on the assumption of Missingness at Random (MAR) [40, 48, 49]. This assumption implies that the probability of a missing variable is dependent solely on observed values. This makes broad sense in the context of this study. For example, whether age is missing for a particular participant is not dependent on their unobserved age. A similar argument can be made for average revenue and the number of days worked by a participant.

In this study, the MICE algorithm is implemented and repeated 10 times to generate 10 imputed datasets in R software. Once this is achieved, a multivariable binary logistic regression is implemented on each complete dataset. For a vector of explanatory study variables $\varvec{X} =\left\{ x_{0}, x_{1}, x_{2},x_{3} \ldots , x_{q}\right\}$ with corresponding coefficients $\varvec{\alpha } =\left\{ \alpha _{0}, \alpha _{1}, \alpha _{2},\alpha _{3} \ldots , \alpha _{q}\right\}$, a multivariable binary logistic regression model is specified as;

1 $$\begin{aligned} \log \bigg (\dfrac{p_{q}}{1-p_{q}}\bigg )=\sum _{q=0}^{Q} \alpha _{q} X_{q}=\varvec{X}^{\prime }\varvec{\alpha } \end{aligned}$$

Here, $p_{i}$ represents the probability of the response being modeled (in our case, 1 denoting a Yes response to medical visit avoidance) that is (i.e., $Y_{i}=1$) for the *i*th study individual. $\log \bigg (\dfrac{p_{i}}{1-p_{i}}\bigg )$ represents the log odds or logit of the probabilities. So, after the $S=10$ imputed and complete datasets are applied to the logistic model, we can denote the estimates and covariances of the model applied to the *S*th completed data set, $(S=1,2,3,4,5,6,7,8,9,10)$ as $\widetilde{\varvec{\alpha }}_{s}$ and $V_{s}$. In this study context, the Multiple Imputation estimate of $\varvec{\alpha }$ is the simple average(or pooling) of all estimates from the 10 datasets applied to binary logistic regression models, given as

2 $$\begin{aligned} \widehat{\varvec{\alpha }}_{\text {MI}}=\frac{1}{10} \sum _{s=1}^{10} \widetilde{\varvec{\alpha }}_{s} . \end{aligned}$$

In addition, the corresponding variance of the MI-estimates can be obtained as;

3 $$\begin{aligned} \widehat{V}_{\text {MI}}=\dfrac{1}{10} \sum _{s=1}^{10} V_{s} + \dfrac{11}{90}\bigg ( \sum _{s=1}^{10}\left( \widetilde{\varvec{\alpha }}_{s}-\widehat{\varvec{\alpha }}_{\text {MI}}\right) \left( \widetilde{\varvec{\alpha }}_{s}-\widehat{\varvec{\alpha }}_{\text {MI}}\right) ^{\prime }\bigg ) . \end{aligned}$$

Based on this information, 95% confidence intervals, *p*-values and associated odds ratio estimates are obtained. All statistical analyses are performed in R software and inferences are made at a 5% significance level.

## Results

### Sample characteristics of the participants and missing data description

**Fig. 1:**
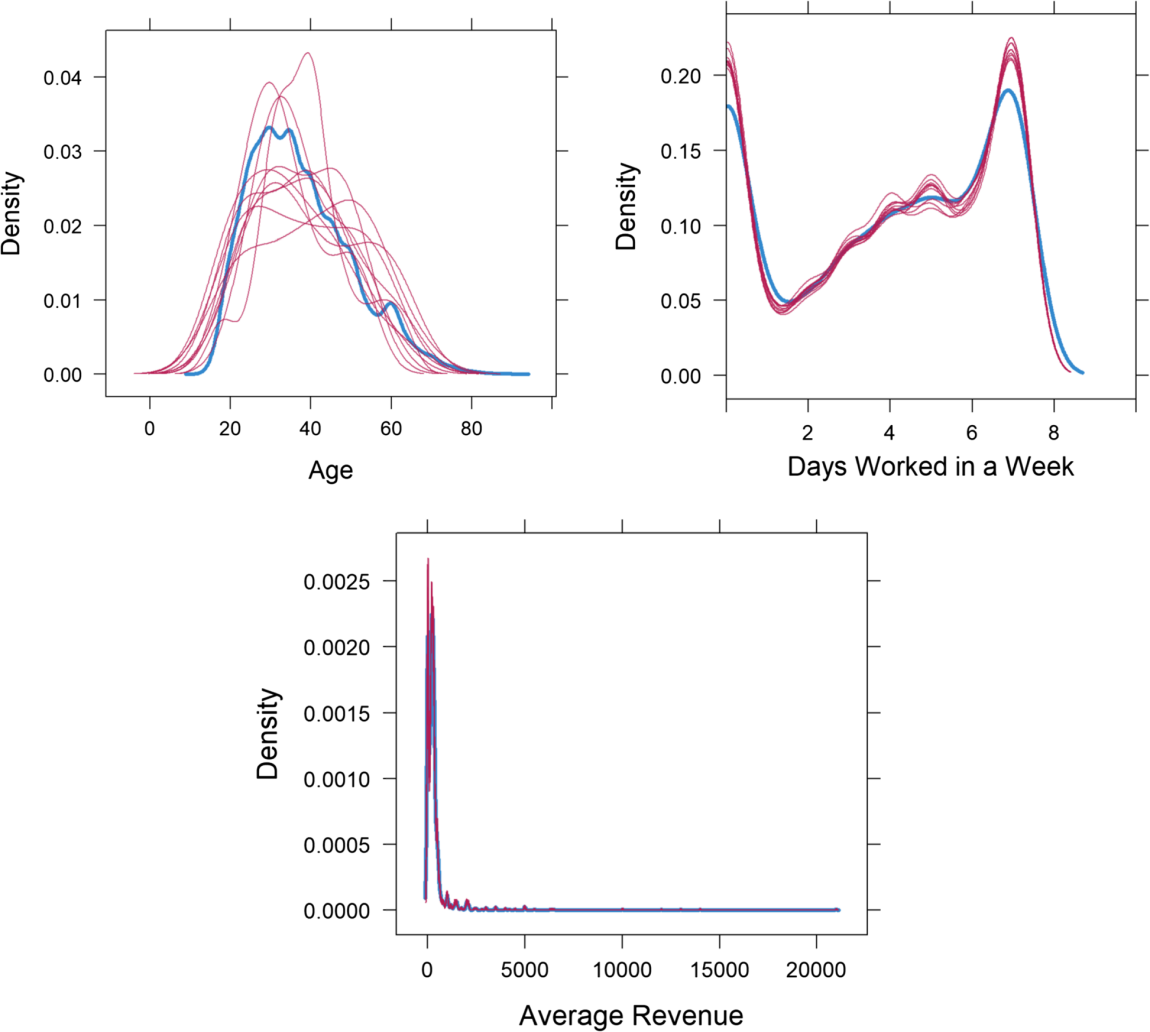
Density plots of multiply imputed variables

Table 1 describes the sample characteristics of the participants. The average age of study participants was 37.7 years with ages ranging from 15 to 88 years.The results further showed that 80.4% of the respondents were males, 19.8% were from the Rajasthan State, 3.6% had class 6-10 education, 42.3% had access to motorcycle transportation, 56.3% received government support, 12.5% used private hospital/clinic, 84.8% were affiliated with the Hinduism religion, 10.8% were daily wage labour in non- agriculture and 15.5% avoided medical visit during the covid-19 pandemic (see Table 1). Regarding the extent of missingness, it was observed that the variables age, average revenue and number of days worked in a week had varying proportions of missing values in the dataset. For example, 69.76% of participants had incomplete profiles due to missing average revenue while 0.11% of participants lacked complete profiles due to age as evidenced in Table 2. Furthermore, to assess the plausibility of imputations, discrepancies between the observed and imputed data are studied via density plots displayed in Fig. 1. This is because the MAR assumption may usually elicit systematic differences between imputed and observed data distributions [50]. In this study, observing Fig. 1, no dramatic differences were observed between the imputed(in red) and observed(in blue) data distributions and as such the imputation models for the variables can be deemed feasible.

**Table 1:** Descriptive statistics of study variables

Variables	Levels/Categories	Sample	Sample Percentage
Gender	Female	920	19.60%
	Male	3762	80.40%
Age (years)	Mean(SD)	37.654(12.444)	
	Range	15.000-88.000	
State	Andhra Pradesh	378	8.10%
	Bihar	942	20.10%
	Jharkhand	919	19.60%
	Madhya Pradesh	859	18.30%
	Rajasthan	928	19.80%
	Uttar Pradesh	656	14.00%
Education	Class 5	26	0.60%
	Class 5 or less	132	2.80%
	Class 6-10	170	3.60%
	High school graduate	111	2.40%
	More than high school	55	1.20%
	No schooling	141	3.00%
	Other	4047	86.40%
Transportation(Via Motorcycle)	No	2702	57.70%
	Yes	1980	42.30%
Government Support (PMGKY)	Received Nothing	2047	43.70%
	Yes	2635	56.30%
Health Facility Type	Anganwadi/ICDS centre	21	0.40%
	Govt Camp	21	0.40%
	Govt. Ayush-related (any)	4	0.10%
	Govt. Dispensary / PHC / CHC	65	1.40%
	Govt. hospital	533	11.40%
	Govt. Mobile clinic	5	0.10%
	NGO or trust hospital/clinic	8	0.20%
	Other private sector facility	24	0.50%
	Other public sector facility	4	0.10%
	Pharmacy/drugstore	61	1.30%
	Pvt ayush-related	10	0.20%
	Pvt. Hospital/clinic	587	12.50%
	Pvt. Mobile clinic	450	9.60%
	Other	2889	61.70%
Medical Visit Avoidance	No	3955	84.50%
	Yes	727	15.50%
Religious Affiliation	Buddhism	47	1.00%
	Christianity	217	4.60%
	Don’t know	16	0.30%
	Hinduism	3968	84.80%
	Islam	330	7.00%
	Jainism	14	0.30%
	Refused to resposnd	8	0.20%
	Sikhism	16	0.30%
	Other	66	1.40%
Primary Work Activity (Primary Source of Income)	Daily wage labour in agriculture	289	6.20%
	Daily wage labour in non-agriculture	505	10.80%
	Did not work for income	724	15.50%
	Other	42	0.90%
	Salaried job in government	42	0.90%
	Salaried job in private company	103	2.20%
	Self-employed in non-cultivation	258	5.50%
	Other	2719	58.10%

**Table 2:** Descriptives on extent of missingness and variables subject to imputation

Variables	Profile	No of Observations	Proportion
^a^Age (years)	Incomplete	5	0.11
	Complete	4677	99.89
^a^Average Revenue	Incomplete	3266	69.76
	Complete	1416	30.24
^a^Days worked in a Week	Incomplete	3317	70.85
	Complete	1365	29.15
State	Incomplete	0	0
	Complete	4682	100
Gender	Incomplete	0	0
	Complete	4682	100
Education	Incomplete	0	0
	Complete	4682	100
Motorcycle Transportation	Incomplete	0	0
	Complete	4682	100
Government Support (PMGKY)	Incomplete	0	0
	Complete	4682	100
Health Facility Type	Incomplete	0	0
	Complete	4682	100
Religious Affiliation	Incomplete	0	0
	Complete	4682	100
Primary Work Activity	Incomplete	0	0
	Complete	4682	100

Note: Variables preceded with ^a^ are those subject to multiple imputation

### Bi-variate analysis of association between socio-demographic factors and COVID-19-induced healthcare utilization avoidance

Table 3 presents a chi-square analysis of the association between socio-economic factors and COVID-19-induced healthcare utilization avoidance. Results showed that gender was significantly related to COVID-19-induced healthcare utilization avoidance. Similarly, each of the independent study variables involving the state of participants, motorcycle transportation, health facility type, and religious affiliation of respondents had statistically significant individual associations with COVID-19-induced healthcare utilization avoidance (see Table 3). Next, inference from the Multivariable Binary logistic regression model via multiple imputation was made.

**Table 3:** Contingency table of medical visit avoidance and independent categorical study variables

Variables	Levels/Categories	Medical Visit Avoidance		Chi-Square Test
		No	Yes	*P*-value
Gender	Female	82.17% (756)	17.83% (164)	0.036
	Male	85.03% (3199)	14.97% (563)	
State	Andhra Pradesh	86.24% (326)	13.76% (52)	0.0076
	Bihar	77.49% (730)	22.51% (212)	
	Jharkhand	88.14% (810)	11.86% (109)	
	Madhya Pradesh	80.56% (692)	19.44% (167)	
	Rajasthan	90.09% (836)	9.91% (92)	
	Uttar Pradesh	85.52% (561)	14.48% (95)	
Education	Class 5	84.62% (22)	15.38% (4)	0.6677
	Class 5 or less	86.36% (114)	13.64% (18)	
	Class 6-10	83.53% (142)	16.47% (28)	
	High school graduate	83.78% (93)	16.22% (18)	
	More than high school	78.18% (43)	21.82% (12)	
	No schooling	88.65% (125)	11.35% (16)	
	Other	84.41% (3416)	15.59% (631)	
Age (years)	Mean (SD)	37.788 (12.455)	36.921 (12.368)	
	Range	15.000 - 88.000	15.000 - 85.000	
Transportation(Via Cycle)	No	87.48% (2626)	12.52% (376)	0.0004
	Yes	84.03% (1847)	15.97% (351)	
Government Support (PMGKY)	Received Nothing	86.08% (2326)	13.92% (376)	0.2431
	Yes	82.27% (1629)	17.73% (351)	
Health Facility Type	Anganwadi/ICDS centre	80.95% (17)	19.05% (4)	$<0.0001$
	Govt Camp	85.71% (18)	14.29% (3)	
	Govt. Ayush-related (any)	50.00% (2)	50.00% (2)	
	Govt. Dispensary / PHC / CHC	78.46% (51)	21.54% (14)	
	Govt. hospital	79.36% (423)	20.64% (110)	
	Govt. Mobile clinic	80.00% (4)	20.00% (1)	
	NGO or trust hospital/clinic	75.00% (6)	25.00% (2)	
	Other private sector facility	87.50% (21)	12.50% (3)	
	Other public sector facility	50.00% (2)	50.00% (2)	
	Pharmacy/drugstore	86.89% (53)	13.11% (8)	
	Pvt ayush-related	70.00% (7)	30.00% (3)	
	Pvt. Hospital/clinic	75.13% (441)	24.87% (146)	
	Pvt. Mobile clinic	77.11% (347)	22.89% (103)	
	Other	88.72% (2563)	11.28% (326)	
Religious Affiliation	Buddhism	91.49% (43)	8.51% (4)	0.0116
	Christianity	89.86% (195)	10.14% (22)	
	Don’t know	93.75% (15)	6.25% (1)	
	Hinduism	83.80% (3325)	16.20% (643)	
	Islam	88.48% (292)	11.52% (38)	
	Jainism	85.71% (12)	14.29% (2)	
	Refused to resposnd	75.00% (6)	25.00% (2)	
	Sikhism	62.50% (10)	37.50% (6)	
	Other	86.36% (57)	13.64% (9)	
Primary Work Activity(Source)	Daily wage labour in agriculture	80.97% (234)	19.03% (55)	0.4324
	Daily wage labour in non-agriculture	85.94% (434)	14.06% (71)	
	Did not work for income	86.60% (627)	13.40% (97)	
	Other	88.10% (37)	11.90% (5)	
	Salaried job in government	85.71% (36)	14.29% (6)	
	Salaried job in private company	84.47% (87)	15.53% (16)	
	Self-employed in non-cultivation	87.21% (225)	12.79% (33)	
	Other	83.67% (2275)	16.33% (444)	

### Multivariable binary logistic regression analysis of association between socio-demographic factors and COVID-19-induced healthcare utilization avoidance

The multivariable binary logistic regression via multiple imputation is presented in Table 4. Respondents living in Bihar state were 2.14 times (OR=2.14; 95%CI=1.47, 3.11), more likely to avoid a medical visit due to COVID-19 compared to respondents living in Andhra Pradesh. Also, residents in Madhya Pradesh were 70% (OR=1.70; 95%CI=1.17, 2.47) more likely to avoid a medical visit due to COVID-19 relative to their counterparts in Andhra Pradesh. Furthermore, the likelihood of Rajasthan residents to avoid a medical visit due to COVID-19 was 32% (OR=0.68; 95%CI=0.46, 1.01) less than residents in Andhra Pradesh. Respondents who had had more than a high school education were about 3 times (OR=3.09; 95%CI=1.31,7.29) more likely to avoid a medical visit due to COVID-19 in comparison with those without any form of schooling record. In addition, regarding the effect of government support under PMGKY scheme on medical visit avoidance due to COVID-19, there was not a statistically significant association between those who received support or not and their tendency to avoid a medical visit. Additionally, participants who utilized a government dispensary and a government hospital were 2.53(OR=2.53; 95%CI=0.96,6.71) and 1.93 (OR=1.93; 95%CI=0.88,4.25) times respectively more likely to avoid a medical visit due to COVID-19 compared to those patronizing a pharmacy or drugstore. Furthermore, the odds for private hospital users to avoid a medical visit due to COVID-19 were  2.35 times (OR=2.35; 95%CI=1.08,5.13) higher relative to users of a pharmacy. Pertaining to religious affiliation, the odds of being a part or not of any kind of religious sect did not significantly influence the probability to avoid a medical visit due to COVID-19.(see Table 3).

**Table 4:** Multiple binary logistic regression results via multiple imputation

Variable	OR	*P*-value	Lower	Upper
(Intercept)	0.1426	0.0421	0.0218	0.9324
Age (years)	0.9955	0.2032	1.2253	1.0024
State(Ref=Andhra Pradesh)				
Bihar	2.1389	0.0001	1.4715	3.1091
Jharkhand	0.8931	0.5561	0.6128	1.3015
Madhya Pradesh	1.7020	0.0051	1.1735	2.4685
Rajasthan	0.6824	0.0574	0.4601	1.0121
Uttar Pradesh	1.2021	0.3834	0.7946	1.8187
Days worked in a Week	1.0099	0.6231	0.9701	1.0514
Average Revenue	1.0000	0.4940	0.9999	1.0001
Gender(Ref=Female)				
Male	0.8248	0.0716	0.6688	1.0171
Education(Ref=No Schooling)				
Refused to Disclose	1.4186	0.2056	0.8254	2.4379
Class 5	2.0875	0.2369	0.6165	7.0687
Class 5 or less	1.3828	0.3919	0.6583	2.9047
Class 6-10	1.6557	0.1456	0.8395	3.2653
High school graduate	2.0385	0.0638	0.9601	4.3284
More than high school	3.0942	0.0097	1.3141	7.2858
Motorcycle transportation(Ref=No)				
Yes	0.8749	0.1982	0.8413	1.0725
Government Support (PMGKY) (Ref=Received Nothing)				
Yes	0.9976	0.9783	1.0278	1.1830
Health Facility Type(Ref=Pharmacy/drugstore)				
Refused to disclose	0.8958	0.7782	0.4165	1.9266
Anganwadi/ICDS centre	1.6280	0.4759	0.4263	6.2170
Govt Camp	1.3412	0.6931	0.3119	5.7666
Govt. Ayush-related (any)	6.9451	0.0768	0.8115	59.4411
Govt. Dispensary / PHC / CHC	2.5333	0.0614	0.9565	6.7098
Govt. hospital	1.9304	0.1020	0.8776	4.2462
Govt. Mobile clinic	1.9749	0.5698	0.1888	20.6557
NGO or trust hospital/clinic	2.5077	0.3130	0.4203	14.9629
Other private sector facility	0.7525	0.7007	0.1764	3.2095
Other public sector facility	7.6378	0.0666	0.8699	67.0618
Pvt ayush-related	2.8415	0.1945	0.5865	13.7671
Pvt. Hospital/clinic	2.3512	0.0318	1.0774	5.1311
Pvt. Mobile clinic	1.8638	0.1218	0.8470	4.1016
Religious Affiliation(Ref=Jainism)				
Other	0.6976	0.6821	0.1245	3.9098
Buddhism	0.2884	0.1961	0.0438	1.9000
Christianity	0.4733	0.3689	0.0925	2.4206
Don’t know	0.1812	0.1947	0.0137	2.3965
Hinduism	0.6307	0.5624	0.1325	3.0008
Islam	0.4645	0.3461	0.0942	2.2902
Other	1.6669	0.6559	0.1760	15.7867
Sikhism	2.4376	0.3547	0.3692	16.0922
Primary Work Activity(Ref=Did not work for income)				
Refused to disclose	1.3555	0.0216	1.0457	1.7571
Daily wage labour in agriculture	1.5069	0.0377	1.0236	2.2184
Daily wage labour in non-agriculture	1.0294	0.8729	0.7219	1.4678
Other	0.8946	0.8259	0.3317	2.4133
Salaried job in government	1.0980	0.8472	0.4240	2.8437
Salaried job in private company	1.2885	0.4179	0.6976	2.3800
Self-employed in non-cultivation	1.0385	0.8709	0.6582	1.6387

## Discussions and conclusions

### Main findings

This study has highlighted six key important findings for possible interpretation and policy implications. First, the prevalence of COVID-19-induced healthcare utilization avoidance was 15.5% in rural India. Second, participants from the Bihar State have significantly higher likelihood of COVID-19-induced healthcare utilization avoidance compared to those from the Andhra Pradesh. Third, participants with more than high school education have significantly higher odds of COVID-19-induced healthcare utilization avoidance compared to those with no schooling. Fourth, participants who used private hospital/clinic significantly have higher odds of COVID-19-induced healthcare utilization avoidance compared to those who visit the pharmacy/drugstore. Fifth, participants with engaged in agriculture have significantly higher odds of COVID-19-induced healthcare utilization avoidance compared to those who did not work for income. These findings have been discussed in relation to previous studies. Also, the policy, practice and research implications have further been highlighted for the attention of policy makers, consumers of healthcare during COVID-19 pandemic in rural India, and health researchers in general.

### Interpretation of the findings in relation to previous studies

COVID-19-induced healthcare utilization avoidance is an important policy health issue which needs to be given much attention in both policy and research discussions. Despite its importance to policy decision, not much is known about demographic and socio-economic factors explaining COVID-19-induced healthcare utilization avoidance in rural India. Drawing evidence from a representative sample in rural India, the objectives of the study are: 1) to estimate the prevalence of COVID-19-induced healthcare utilization avoidance in rural India 2) to determine if demographic and socio-economic factors predict COVID-19-induced healthcare utilization avoidance in rural India. The study revealed that the prevalence of COVID-19-induced healthcare utilization avoidance was 15.5%. Although, there are no indicators/measurements/scales to enable the authors to determine whether our prevalence of COVID-19-induced healthcare utilization avoidance is low, moderate, or high, based on the few available literature, we argue that the prevalence of COVID-19-induced healthcare utilization avoidance reported in this study in rural India is low compared to 20.2% rate in population-based Rotterdam study [27], 33.3% reported rate among adults in the United States [51] and 73.2% rate among the general population in South Korea [52]. This finding underscores the fact that there has been a decline in non-COVID-19 healthcare utilization due to the COVID-19 pandemic [53, 54]. The disparities in COVID-19-induced healthcare utilization avoidance between this current study and previous studies could be attributed to geographical location, rate of COVID-19 infections and deaths, sample size, the unit of analysis, healthcare infrastructure, and conceptualization of healthcare utilization in rural India and United States. For instance, whereas our study was limited to rural people in India, Czeisler et al’s [51] study was focused on both rural and urban areas hence accounting for differences in the prevalence of COVID-19-induced healthcare utilization avoidance.

Prior to the data collection, India had 5,992,532 confirmed COVID-19 cases and 94,503 deaths [55], but with relatively low COVID-19-induced healthcare utilization avoidance. Beyond the other reasons espoused, in North India, a rural cohort study reported formal healthcare utilization in pre-COVID-19 times to be 79% (with 21% healthcare utilization avoidance rate) [56], while healthcare pre-COVID-19 healthcare utilization was found to be 88.99% (with 11.01% healthcare utilization avoidance rate) among older adults (60 years and above) [57].

A superficial inference from this suggests a non-significant difference between pre-COVID-19 and COVID-19-induced healthcare utilization avoidance. The relaxation of lockdown measures (lifting of national lockdown months prior to the study, although localized lockdowns were continued in hotspot areas) [58], might have also influenced the health behaviour of the participants (motivating them to resume regular activities, including attending to their healthcare needs from health facilities - but with care and vigilance, and abiding by other health measures). Although rural folks have a more limited access to health services [59], some studies suggest that on the contrary, living in rural area could be a protective factor at least during the first phase of the pandemic [60, 61] due to lower population density, a factor associated with lower prevalence and incidence of infection [59]. Upon this assumption, the impacts of COVID-19 with regards to disruptions to the provision of healthcare services could be minimal, leading to insignificant healthcare utilization avoidance from pre-COVID-19 times. Although fear and avoidance of healthcare workers is a widespread, under-recognized problem during the COVID-19 pandemic [62], the changing information about the virus [63] could have led to instances where people presume any health issue to be COVID-19-related. Again, Nilima et al. [28] discovered that perceived threat to the well-being of their loved ones had a substantial impact on people’s adherence to the lockdown measures, which possibly could decrease individuals’ healthcare-seeking behavior, potentially leading to avoidance of healthcare services during the COVID-19 pandemic. Situations like this could increase health service utilization (at least temporarily) for people who desire to get tested and seek treatment, thus inadvertently reducing COVID-19-induced healthcare utilization avoidance.

Geographic region can significantly influence the prevalence of infectious diseases through the interplay of climate, environment, biodiversity, socioeconomic factors, healthcare infrastructure, and population mobility. These factors interact to shape the prevalence of COVID-19 in different geographic regions [31, 64], highlighting the importance of considering the local context and implementing tailored strategies for effective disease control and mitigation.

The study has established an association between State of residence and COVID-19-induced healthcare utilization avoidance. Consistent with our findings, in South Korea a relationship has been established between residential area and COVID-19-induced healthcare utilization avoidance [52]. More importantly, we found that participants from the Bihar State and Madhya Pradesh State have significantly higher likelihood of COVID-19-induced healthcare utilization avoidance compared to those from the Andhra Pradesh, while participants from Rajasthan State have significantly lower likelihood of COVID-19-induced healthcare utilization avoidance compared to those from the Andhra Pradesh. Since this finding is new in the COVID-19-induced healthcare utilization avoidance literature, the authors were not able to get more studies to support their findings. Regardless of this, the reasons for the differential COVID-19-induced healthcare utilization avoidance among the various States of residence in rural India could be assigned to rate of COVID-19 cases and the number of health facilities. For instance, all things being equal, in States where the rate of COVID-19 cases is high, people may be less likely to go out to seek non-COVID-19 healthcare services because of fear of contracting the virus.

According to data from Rajasthan’s Department of Medical Health and Family Welfare, as of September 2020, the state’s COVID-19 cumulative cases were 113,124 (with 1,322 death) [65], significantly lower than that of Andhra Pradesh (687,351 positive cases, 59,435 active cases and 5,780 deaths).

For instance, Lee & You [52] have argued that people who are living in highly affected residential areas have higher odds of avoiding healthcare use during the COVID-19 pandemic. Also, COVID-19 may exacerbate pressure on the existing facilities which could subsequently impact on the utilization of non-COVID-19 healthcare services [66].

For illustration, Middle East respiratory syndrome coronavirus (MERS-CoV) epidemic occurred in Korea in 2015, with the majority of cases being hospital-acquired infections [67]. As a result, it is assumed that people may be worried about contracting the virus from visiting hospitals. Additionally, healthcare resources were also directed toward treating individuals with severe COVID-19 in places where the disease was endemic [68], leaving non-COVID-19 healthcare services unattended to [69]. This however did not hold in all instances, as Bihar with a cumulative COVID-19 case of 104,093 (with 537 deaths) and Madhya Pradesh with COVID-19 case count of 93,053 (with 1,820 deaths) had significantly higher COVID-19-induced healthcare utilization avoidance compared to Andhra Pradesh with a total positive case of 6,87,351 (including 59,435 active cases and 5,780 deaths). Such a situation could stem form localized extension of lockdowns as in the case of Bihar where the state government decided to extend the lockdown till September 6, owing to the rising cases of COVID-19 in the state. The lifting of lockdown measures in this state at a latter date, compared to Andhra Pradesh might have also influenced explaining COVID-19-induced healthcare utilization avoidance. Furthermore, in contrast to Andhra Pradesh, where it is clear that creative measures have been taken by the state government to increase access to high-quality healthcare [70], the health system in Bihar is far from ideal due to a significant shortfall of facilities and staff [71]. This disparity could have created pre-COVID-19 healthcare utilization gaps between these two states, partly in favor of Andhra Pradesh, where accessibility is higher [70, 71] with the COVID-19 pandemic only exacerbating these gaps.

The study revealed a relationship between educational level and COVID-19-induced healthcare utilization avoidance. Our findings are not consistent with a previous study on avoidance of healthcare utilization in South Korea during the COVID-19 pandemic which showed no association between education and avoidance of healthcare utilization [52]. However, the findings from our study are consistent with a national household survey in Peru which revealed an association between education and non use of health services [72]. This suggests that there is mixed evidence on the linkage between education and COVID-19-induced healthcare utilization avoidance [27, 52, 72]. More importantly, our findings advance knowledge that participants with more than a high school education have significantly higher odds of COVID-19-induced healthcare utilization avoidance compared to those with no form of formal education. This finding could be attributed to the fact that people with higher level of education have significant knowledge, positive attitudes, and good practices regarding the preventions of spread of COVID-19 pandemic [73], resulting in higher likelihood of avoiding healthcare services during the COVID-19 pandemic.

The study has further established that type of healthcare facility and employment type are associated with COVID-19-induced healthcare utilization avoidance in rural India. Specifically, we found that participants who utilise private hospital/clinic and those who receive daily wage from agriculture (agriculture as primary economic activity) are more likely to avoid healthcare utilization during COVID-19 pandemic. These findings underscore the role of type of healthcare facility and type of work or source of income in explaining COVID-19-induced healthcare utilization avoidance. The disparities in the COVID-19-induced healthcare utilization avoidance among the various work or employment groups could be attributed to differences in health literacy, healthcare accessibility and affordability in rural India. For instance, the literature is replete with low health literacy among farmers and farming households [74, 75] and low levels of healthcare utilization due to poverty [76–78]. The implications for policy, practice and research are therefore highlighted.

### Implications for policy, practice, research and limitations

The study has contributed to literature in relation to prevalence of COVID-19-induced healthcare utilization avoidance and associated demographic and socio-economic factors. These findings are important in bridging the COVID-19-induced healthcare utilization avoidance information gap(s) in rural India. The findings have highlighted some key areas for practice, policy and research which are integral in reducing the prevalence of COVID-19-induced healthcare utilization avoidance in rural India. Thus, the findings from the study offer important contributions to practice, policy and research. From the practice perspective, the findings suggest the need to educate and sensitize the participants regularly (every 3 months) using various media platform (such as television and radio, religious gatherings, local/community leadership meetings etc.) to frequently seek non-COVID-19 healthcare services. The recommendation to sensitize participants every three months is based on several justifications. First, regular sensitization will ensure that participants receive updated information and guidance as circumstances change, particularly in dynamic situations like a pandemic. Second, it will help reinforce knowledge and behavioral practices over time, combating forgetfulness and competing priorities. Lastly, spacing out sensitization sessions every three months is to help overcome information fatigue, ensuring that participants can engage with and absorb the information effectively. We believe that when education is done every 3 months, a balance between information adequacy, underload, and overload will be achieved. Since overload of information may impede the decision-making process, resulting in a poor (or even no) decision being made, information underload may not empower people to take preventative action. By considering these factors, regular sensitization can maximize the chances of sustained behavior change and promote informed decision-making among participants.

In rural India, sensitization efforts should be tailored to the specific context and challenges of reaching rural populations. Strategies such as community-based workshops, localized information campaigns, engaging local influencers, mobile messaging and voice calls, and integration with existing community programs can be effective. The approach should aim to utilize community resources, address language and literacy barriers, and leverage mobile phone networks to disseminate information in a culturally sensitive and accessible manner. By engaging with local communities, utilizing existing networks, and embracing technology, sensitization efforts can effectively reach and empower rural populations in India during pandemics. Such education should be spearheaded by health officials such as doctors, nurses, and midwives to receive participation from healthcare users in rural India. This education may well help to eliminate or minimise fears or psychological distress in people to ensure higher healthcare utilization during the COVID-19 pandemic in rural India.

We, however, acknowledge that education alone may not completely eliminate fear or distress during pandemics or public health emergencies. Fear and distress affect individuals across various educational backgrounds and professions. Nevertheless, education and awareness campaigns can play a significant role in providing accurate information, dispelling myths, and promoting preventive measures. By enhancing health literacy, education initiatives can empower individuals to make informed decisions, adopt recommended behaviors, and understand the rationale behind public health measures. While education may not eliminate fear entirely, it can contribute to a better understanding of the situation and help individuals manage their emotions and responses more effectively. To effectively address future pandemics and public health emergencies, there is the need for strengthening healthcare infrastructure in rural areas, improving health literacy and public awareness through education campaigns, enhancing community engagement and participation, ensuring equitable access to healthcare services, investing in healthcare workforce training and capacity-building, establishing robust surveillance and monitoring systems, fostering interdisciplinary collaboration, supporting mental health and psychosocial well-being, promoting research and data collection, and leveraging mHealth options. Utilizing mobile health technologies can lessen barriers to healthcare during crises by eliminating physical contacts, enabling remote consultations, providing access to information and resources, facilitating contact tracing, and supporting self-monitoring and self-care. Integrating mHealth solutions into emergency response strategies can also enhance healthcare delivery, improve communication, and mitigate the impact of pandemics and public health emergencies on healthcare utilization avoidance. From the policy perspective, policy makers should consider the inclusion of specific demographic and socio-economic variables such as State of residence, educational level, type of public health facility and employment type in the formulation of policy aimed at scaling down COVID-19-induced healthcare utilization avoidance in rural India. This is because, the above demographic and socio-economic variables were associated with COVID-19-induced healthcare utilization avoidance in rural India. From a research perspective, due to the quantitative nature of the dataset used in this study which we hereby highlight as a potential limitation, we only considered demographic and socio-economic variables predicting COVID-19-induced healthcare utilization avoidance without inclusion of health-related and lifestyle variables such as self-rated health, psychological distress (mental distress), physical activity, smoking, alcohol consumption, fruits, and vegetable intakes which are all important variables for measuring healthcare utilization avoidance. For this same reason, future studies should extend further the variables considered in this study by including health-related and lifestyle variables. For instance, further study could examine demographic, socio-economic, health-related and lifestyle factors explaining COVID-19-induced healthcare utilization avoidance in rural India.

More importantly, the quantitative nature of the study limited us to qualitatively capture the normative views and standpoint of the participants in relation to COVID-19-induced healthcare utilization avoidance in rural India. The absence of qualitative insights deprives the study of a more nuanced understanding of participants’ perspectives and experiences. Due to the quantitative inclination of this study, which is acknowledged as a limitation of our study, a mixed methods study on COVID-19-induced healthcare utilization avoidance in rural India and elsewhere is welcomed. The findings from this study further offer opportunity for research on COVID-19-induced healthcare utilization avoidance in rural and urban communities in India. Such research would help to determine geographical disparities (that is rural vs. urban) in terms of the prevalence of COVID-19-induced healthcare utilization avoidance and associated factors in India. The findings from the above proposed study may well help to implement location specific measures/strategies to reduce COVID-19-induced healthcare utilization avoidance in India and other countries which share similar characteristics with the population of rural India. Aside from the limitations which have been integrated into the preceding paragraph, we could not determine a causal relationship between our independent variables (demographic and socio-economic factors) and dependent variable (COVID-19-induced healthcare utilization avoidance) because of the cross-sectional nature of the study. However, the purpose of this study was not to draw any causal relationship between the independent variables and the dependent variables, we were only interested in establishing if demographic and socio-economic variables predict COVID-19-induced healthcare utilization avoidance in rural India. Thus far, future studies interested in drawing causal relationships should consider a longitudinal study. Furthermore, measuring the dependent variable which is COVID-19-induced healthcare utilization avoidance as a dichotomous variable could be another potential limitation of the study. Lastly, temporal limitations and the dynamic nature of the COVID-19 pandemic suggest that the prevalence of healthcare utilization avoidance and associated factors may evolve over time, warranting further investigation. Despite these limitations, the study contributes valuable insights into the phenomenon of COVID-19-induced healthcare utilization avoidance in rural India, serving as a foundation for future research endeavors to address these limitations and advance our knowledge of healthcare-seeking behaviors during public health emergencies.

## Data Availability

The data that support the findings of this study are available from the World Bank, but restrictions apply to the availability of these data, which were used under license for the current study and so are not publicly available. Data are, however, available from the authors upon reasonable request and with permission of the World Bank.
